# Responding to the Current Psychedelics Landscape: A Call for Cross-Sector Collaboration

**DOI:** 10.1017/jme.2025.10110

**Published:** 2025

**Authors:** Lori Bruce

**Affiliations:** Interdisciplinary Center for Bioethics, Yale University, New Haven, United States

**Keywords:** psychedelics, ethics, bioethics, deregulation, psilocybin, harm reduction

## Abstract

Psychedelics are becoming increasingly available within approved regulatory pathways and in “underground” or recreational settings. However, clinicians’ knowledge and training is insufficient, leading to limitations when discussing benefits and harms with patients. These insufficiencies also create liability risks for clinicians which may be heightened if, as anticipated, the federal government deregulates psychedelics. In light of rapidly changing conditions, stakeholders should work together to increase public and clinical education. Stakeholders should also develop pathways for widely available post-trip counseling services. Such pathways should address the needs of users struggling to process the ongoing emotional and neuropsychiatric effects of their psychedelics experience which can sometimes be disabling. Thoughtful and timely collaboration can lay the groundwork for psychedelic medicine, a newly developing area of clinical practice.

Cheung et al.’s article in this month’s issue discusses the risks of medical liability for physicians prescribing psilocybin or facilitating psilocybin treatment.^1^ The authors outline a number of contributing factors to liability including gaps within medical training, standards, and credentialing. These components are necessary to maximize therapeutic potential, reduce harms, and foster access. The gaps they describe may also compromise clinician-patient relationships as clinicians may feel unprepared for — and at-risk from — frank conversations with their patients about psychedelics.

Many research subjects have experienced profound outcomes with psychedelics. However, a number of factors raise skepticism about the generalizability of some psychedelic clinical trials. Methodological concerns include overly stringent subject selection and inconsistent reporting of harms.^2^ Additionally, some psychedelics users (in both clinical trials and recreational use) have experienced harms from their use of psychedelics. A small percentage have developed PTSD, anxiety, and depression *from* their psychedelics use, leading Evans to refer to psychedelics as a “double-edged sword.”^3^

The field has long been aware that harm reduction methods can promote good outcomes. Careful preparation and mindset (referred to as “set and setting”) enables the psychedelics user to prepare for the unique and sometimes unsettling aspects of these substances.^4^ However we still lack definitive findings on who is more likely to suffer harms, and a small percentage suffer from long-term adverse effects. These include depersonalization and derealization in which users may feel they have lost control of their actions, thoughts, and body or may perceive reality as unreal, as if they are in a movie.^5^ Others suffer from severe sleep impairment and anhedonia, even in the context of “multiple psychological, social, and environmental protective factors.”^6^ Without greater awareness of which users are more likely to suffer harms, the consent process experiences limitations.^7^

We are entering a period of increased access and decreased federal regulation.^8^ Deregulation can reduce barriers to access, reduce costs, foster innovation, and stimulate market growth. However, deregulation may also fail to protect psychedelics users and indigenous communities from corporate abuse.^9^ The state level is also seeing changes to psychedelics access and criminalization. This year, over 60 psychedelics bills were introduced within 22 US states.^10^ Arizona proposed a complex regulated process similar to Oregon’s current model.^11^ Other states proposed down-scheduling of psychedelics^12^ or milder penalties for possession of small amounts of psilocybin.^13^ However these state bills progress, federal deregulation calls for action.

Work will fall on state governments, medical associations, the research community, and other stakeholders. Their success will depend on time-sensitive cross-sector collaboration. These parties should promote studies identifying who is more likely to be at risk for long-term adverse outcomes — and who is more likely to benefit from psychedelics. Cross-sector collaboration should also develop freely accessible online public education materials helping potential users understand benefits and risks of psychedelics.

Education should provide drug-specific guidance (e.g., when users may benefit from psilocybin versus MDMA or ibogaine) along with guidance on choosing a facilitator, seeking integration support, and recovering from adverse effects. Psychedelics users should be especially aware of the wide range of beliefs held by facilitators and integration specialists. Some integration specialists, for example, believe adverse effects are not harmful and should be welcomed as part of the healing process.^14^ Others may discourage users from seeking medical advice.^15^ Psychedelics users must understand how to screen for assistance in a way that is both supportive of evidence-based medicine and aligned with their views and values. Otherwise, they risk further delays in care.

Educational materials developed by cross-sector collaboration can also be useful for clinicians to increase their understanding of psychedelics and promote nonjudgmental patient guidance on harm reduction, benefits, and consent. Perhaps most importantly, cross-sector collaboration must address the needs of users who are suffering from adverse effects or otherwise struggling to process the ongoing emotional and neuropsychiatric effects of their psychedelics experience. This calls for accessible, safe integration and recovery services, perhaps with a free and nonjudgmental “drop-in” format offered online to reach the widest audience. The field must also develop robust treatment protocols for users experiencing adverse effects with insights from recent literature.^16^

In closing, this commentary outlines a number of considerations as psychedelics become more available within the US, Canada, and across the globe. Work must be done to facilitate clinicians’ ability to offer meaningful patient guidance on psychedelics while decreasing their risks of medical liability. If enacted, Cheung et al.’s proposals, along with work outlined above, will further these efforts. Given the rapid pace of deregulation seen in the US, and the time required to construct a proper safety and educational infrastructure, clinicians should proceed with caution. Funders should consider supporting the work highlighted above and outlined by Cheung et al.^17^ This would further funders’ goals while promoting a safer, more transparent environment. Without quick and thoughtful action, increased access to psychedelics could increase harms and unintended consequences. This could backfire and lead to across-the-board restrictions in psychedelics access to prevent additional harms. To prevent such a pendulum effect, stakeholders must work together with haste and care.
